# Bare Soil Surface Moisture Retrieval from Sentinel-1 SAR Data Based on the Calibrated IEM and Dubois Models Using Neural Networks

**DOI:** 10.3390/s19143209

**Published:** 2019-07-21

**Authors:** Hamid Reza Mirsoleimani, Mahmod Reza Sahebi, Nicolas Baghdadi, Mohammad El Hajj

**Affiliations:** 1 Faculty of Geodesy and Geomatics Engineering & Remote Sensing Institute, K. N. Toosi University of Technology, Tehran 19667-15433, Iran; 2 IRSTEA, UMR TETIS, University of Montpellier, 500 rue François Breton, 34093 Montpellier cedex 5, France

**Keywords:** bare soils, soil moisture, neural networks, Sentinel-1, calibrated IEM, Modified Dubois Model, Iran

## Abstract

The main purpose of this study is to investigate the performance of two radar backscattering models; the calibrated integral equation model (CIEM) and the modified Dubois model (MDB) over an agricultural area in Karaj, Iran. In the first part, the performance of the models is evaluated based on the field measurement and the mentioned backscattering models, CIEM and MDB performed with root mean square error (RMSE) of 0.78 dB and 1.45 dB, respectively. In the second step, based on the neural networks (NNS), soil surface moisture is estimated using the two backscattering models, based on neural networks (NNs), from single polarization Sentinel-1 images over bare soils. The inversion results show the efficiency of the single polarized data for retrieving soil surface moisture, especially for VV polarization.

## 1. Introduction

Soil surface parameters, especially soil moisture, are the key parameters for numerous agricultural and hydrological applications [1,2]. Synthetic aperture radar (SAR) data have been widely used for long-term soil parameters monitoring over large areas [3,4,5,6,7]. In this regard, the sensitivity of SAR signal to soil moisture benefits water management, flood forecasting, drought monitoring, and sustainable agriculture. Currently, because of free and open access to high resolution and high revisit time C-band SAR data, Sentinel-1 constellation can offer great potential to improve the estimation of soil surface parameters.

The backscattering coefficient of SAR systems is the function of physical and electrical properties of a target [8,9,10,11] and SAR configuration (frequency, incidence angle, and polarization). Soil surface moisture and soil roughness are the two most important soil parameters affecting the backscattering coefficient [12]. It is frequently argued that the effects of soil surface roughness are equal or greater than those of soil moisture content on the backscattering coefficient [13,14,15].

To date, several studies have assessed the bare soil surface parameters estimation by physical and empirical backscattering models, using SAR data. Unlike empirical models, a physical model does not need any specific site calibration. The most commonly used physical model is the IEM (integral equation model) developed by Fung and Chen (1992) [16]. In the IEM, *σ*^0^ is considered as a function of SAR configuration and soil parameters. Roughness parameters are the root mean square of height (*h_rms_*), which is the vertical standard deviation of the roughness, and the correlation length (*L*) represents the horizontal roughness scale. To improve the IEM accuracy, [17,18,19,20] have proposed an IEM’s semi-empirical calibration (CIEM) model by reforming *L* with the calibrated parameters, called *Lopt*. *Lopt* depends on SAR configuration and *h_rms_* [21]. So far, three different function shapes, namely, linear, exponential, and power, have been utilized to compute *Lopt* from the SAR parameters and *h_rms_*. The Gaussian function shape provides the best accuracy on simulated *σ*^0^ from the modified IEM. The IEM modified by [17,18,19,20,21] (CIEM) was evaluated by using a large dataset at X- and C-bands. Their results have shown that the CIEM allows the soil moisture (*M_v_*) estimation with approximately 3 vol.% in the X-band and approximately 6 vol.% in the C-band [3,7,22,23,24,25,26,27]. Recently, Baghdadi et al. (2016) [21] have developed a new model based on Dubois model formulation (MDB) for *M_v_* estimation over bare soils. This model has not been evaluated so far.

In SAR images, the sigma naught (*σ*^0^) which is considered as backscattering coefficient, presents the amplitude of the signal returned from target to SAR antenna that is influenced by the soil surface characteristics which is linked to the soil dielectric constant (soil moisture) and soil surface roughness [28]. Retrieval of soil surface parameters from SAR data normally can be realized using the backscattering model that presents the relation between the target parameters (soil moisture and roughness) and the SAR sensor configurations such as incidence angle, polarization, and frequency [29]. Therefore, it can be definite that the models are engaged to simulate the relation between SAR sigma naught (*σ*^0^) value and sensor-surface parameters. In practice, the estimation of soil surface can be realized by inverting the model while the sensor parameters are known and soil moisture parameter is unknown. However, the main problem is that the radar signal is dependent on both surface roughness and soil moisture parameters. In this regards, we have two unknowns. The classical solution is to use two or more equations, which are multi-configuration SAR data solution [10]. In this approach, we need at least two images with different sensor parameters. The NNs as a machine learning algorithm has the ability to retrieve soil moisture based on the model training using only one image (configuration). In addition, such variables estimation is a nonlinear process and normally multifaceted and complex.

Generally, to invert the models, there are two strategies. First, using the selected model directly; second, using the indirect method. In the first strategy, the main equation will be solved analytically or using numerical algorithms depends on the complexity of the model [10,30]. In the second strategy, based on the training stage, a complex and robust network is engaged. The network, such as the NN and Lookup Tables (LUTs), should be able to present the complexity and all relations of the model [31,32,33]. Because of the complexity of the IEM and CIEM, it is impossible to invert them analytically, therefore, in this study using the NN is considered.

Paloscia et al. in [28,31] have evaluated these inversion techniques using in situ measurements. Because of a reasonable implementation time and acceptable accuracy, the NNs technique is slightly more suitable than the other ones to generate *M_v_* maps. Sahebi et al. (2004) have used the NNs inversion technique to assess soil surface parameters of bare soils from Radarsat-1 SAR data (HH polarization). In this study, the NNs system is trained by two datasets [32]. The first dataset is created using the IEM and the GOM (geometric optics model) [34] and the second one is composed of SAR images and in situ measurements (real dataset). For each dataset, two configurations are considered for NNs training. The first configuration uses backscattering and incidence angles as two inputs. The second configuration uses two different incidence angles as well as the corresponding backscattering coefficients as four inputs. As a result, it was shown that the second configuration provided a better result, considering the trained NNs by the real dataset. Additionally, Baghdadi et al. (2012) [35] have utilized NNs to estimate the soil surface parameters over bare soils. For this case, a synthetic dataset was generated using the CIEM for a wide range of soil conditions (roughness between 0.3 and 3.6 cm and soil moisture between 5 and 45 vol.%). Then, the NNs system was trained by the synthetic dataset and later validated using real SAR data and in situ measurements. In addition, a priori information on the target parameters was considered to improve the accuracy of NNs estimation. The accuracy of the estimation was 7 vol.% for soil moisture and 0.5 cm for soil roughness.

The aim of this study is to evaluate the potential of the CIEM and the MDB for soil moisture (*M_v_*_)_ estimation over the study area in Karaj, Iran. A real dataset has been collected to perform the evaluation. This dataset is divided into two parts, the first part is used to validate the simulation of *σ*^0^ for both models, and the second part is used to estimate the accuracy of *M_v_* by inversion of CIEM and MDB. To estimate *M_v_* from the model, we generated a synthetic dataset of SAR in the C-band (VV and VH polarizations) for a wide range of soil parameters. Then, the neural networks are trained by the synthetic dataset (inputs include one polarization and incidence angle and outputs comprise soil moisture parameters). Afterward, the trained neural networks are used to estimate *M_v_* based on the real SAR observations obtained from Sentinel-1 images. Finally, the estimated *M_v_* are compared with the in situ measurements.

## 2. Study Area and Dataset

### 2.1. Study Area

**Figure 1:**
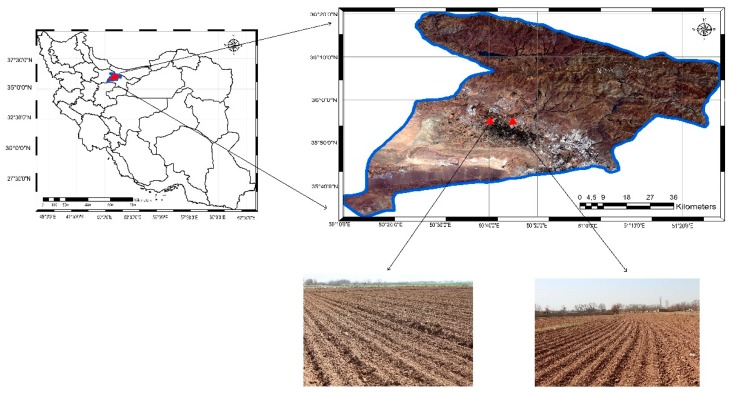
The study area and two examples of the agricultural fields.

The study area is located in Karaj watershed of Iran (latitude = 35° 56′ 34.88” N, longitude = 50° 42′ 27.39” E), covering approximately 100 km^2^ (Figure 1). This area is mostly covered with agricultural fields with diverse cultural types on a relatively flat relief plateau. The properties of soil texture are generally classified as silty clay loam (8% sand, 30% clay, and 62% silt). Additionally, the area experiences a dry climate in summer and a semi-humid to humid one in fall and winter. The diverse climate conditions in this area lead to a different amount of rain and various soil moisture throughout a year. The diverse climate allows cultivating various products. Thus, different levels of roughness have appeared in the study area for which the direction and depth of plow depend on the specific products. As illustrated in Figure 1, some specific areas include bare soils. To be more familiar with the weather of this area, Table 1 presents some weather data for the acquisition data dates.

### 2.2. Satellite Data

In this study, the Sentinel-1 C-band images are used. Based on the period of this satellite and the area’s weather conditions, satellite data acquisition is performed at some specific times and the field measurements are conducted accordingly. In this work, four interferometric wide-swath (IW) images, which are dual-polarization (VV and VH) with a spatial resolution of 10 m, are used. Table 1 summarizes the field measurements and satellite images used in this study.

### 2.3. Field Data

**Figure 2:**
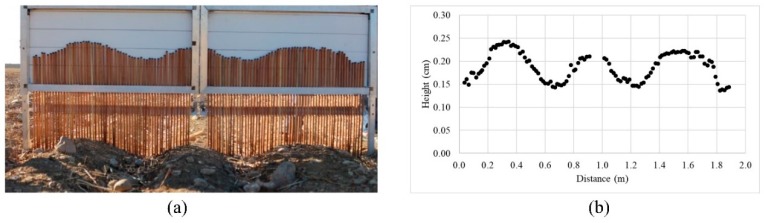
(**a**) A roughness device made in the laboratory; (**b**) an example of its processing (digitizing and calculation) by Webplotdigitizer and ENVI software.

In this research, soil moisture and roughness are conjointly measured the same day as the SAR data acquisitions. Soil surface moisture (*M_v_* in vol.%) was measured in 58 different bare soil land parcels, the areas of which ranged from 2500 to 9000 m^2^. The measurement has been realized with a Thetaprobe sensor based on the time domain reflectometry (TDR) concept. These measurements were achieved for the top of soil (0–5 cm depth), corresponding to the length of the Thetaprobe needles. Using the equation presented in the Thetaprobe soil moisture User Manual (Delta Devices Ltd., 1996), the direct outputs (DC voltage in mV) were converted to soil water content (m^3^m^−3^ and vol.%). The TDR is well calibrated based on the gravimetric method. The number of measurements is adjusted based on the size of each area and soil texture, where at least 30 measurements are conducted for each land parcel. The mean of all measurements was later used for further analysis. The roughness (*h_rms_*) is also measured using a home-made profilometer needle with a length of 2 m and a resolution of 1 cm. (Figure 2). The measurements are parallel and perpendicular to the field furrow. On an average, three parallel and three perpendicular measurements are made for each land parcel. However, the exact number of measurements for each land parcel is based on the roughness non-uniformity of each area. Finally, the roughness value of each area is computed using Equation (1) and the images are processed by ENVI and Webplotdigitizer software packages.

(1) $$h_{rms}=\sqrt{\frac{\sum_{i=1}^{N}\left(z_{i}^{2}-z^{2}\right)}{N-1}}$$

where *N* is the number of profile points, *z_i_* is the surface elevation at point *i* in cm, and *z* is the average surface elevation in cm.

Table 2 shows the main statistical parameters of field measurements. The date of measurements include the different seasons of a year. The first series of measurements were conducted in late summer (21 September 2017) as a dry surface condition. The second series were collected in fall and early winter, when precipitation mostly starts to occur, as a semi-wet condition (19 January 2018). The third series were measured in late winter once it is usually peak of precipitation and soil is often humid (8 March 2018). The time of the last series of measurements were in mid-spring (26 April 2018) when the fields are commonly ready for cultivation and the soil is quite wet due to a high amount of precipitation.

In addition, to define the soil texture, about 20 samples were collected, and then tested in the laboratory. The test result shows that they are composed of about 8% sand, 30% clay, and 62% silt.

## 3. Methodology

**Figure 3:**
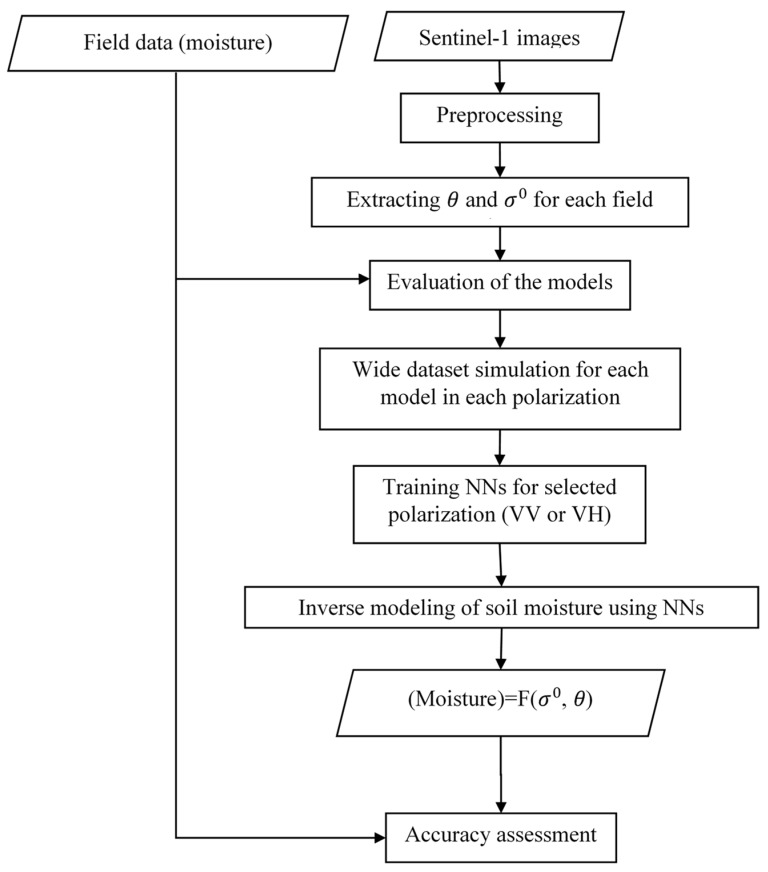
The flowchart of the proposed method (*θ*: incidence angle, *σ*^0^: backscattering coefficient, NNs: neural networks).

The flowchart of the proposed method to retrieve soil moisture is illustrated in Figure 3.

As illustrated in Figure 3, field measurement was realized exactly the same day as satellite data acquisition. After image processing step and calculation of essential parameters for input data for each field (i.e., sigma naught and incidence angle), the work was divided into two steps. In the first step, to evaluate the performance of the model, the measured sigma naught (*σ*^0^) of each field was compared with the simulated sigma naught (*σ*^0^) calculated from each model for both VV and VH polarization. In the second step, soil moisture of each field was estimated from the Sentinel-1 images and then the accuracy was assessed, and the estimated values were compared with the ground measurement. For this step, the NNs were used. The explanation of the phases is discussed in the following subsections.

### 3.1. Satellite Data Preprocessing

The first step of the image processing is preprocessing. The Sentinel Application Platform (SNAP) software is utilized to perform radiometric and geometric corrections. The DN values of raw Sentinel-1 data are first converted to *σ*^0^ using radiometric calibration. Then, the calibrated Sentinel 1 data are georeferenced using the terrain correction algorithm.

In the second step, the mean values of *σ*^0^ are extracted for each field sample. For this, at first, the selected fields were detected on image based on their geographic coordinate. Then the boarder of each field was determined and the average of *σ*^0^ for internal pixels was calculated.

### 3.2. Evaluation of CIEM and MDB

For both models (CIEM and MDB), the variables, including *θ*, *h_rms_* and *M_v_* are obtained from the images and the ground truth measurements are used as known parameters to simulate the *σ*^0^ in VV and VH polarizations. Then, the simulated *σ*^0^ are compared to the same value from Sentinel-1 SAR observation and the accuracy of the simulated values is computed using the RMSE (root mean square error) and the correlation coefficient (R^2^).

### 3.3. Generating Simulated Datasets Using CIEM and MDB

Researchers in [17,18,19] replaced the correlation length of the soil roughness by a range of fitting parameters called *Lopt* which depends on *h_rms_*, *θ,* radar wavelength, and polarization (see Equations (2) and (3) for the C-band). Subsequently, the IEM model is changed to a calibrated IEM by replacing L with *Lopt*. In this study, the calibrated IEM model is used to generate an initial simulated data for the HV and VV polarizations.

(2) $$Lopt\;\left(h_{rms},\theta ,HV\right)\;=\;0.9157\;+\;1.2289\;{\left(\sin0.1543\;\theta \right)}^{-0.3139}\;h_{rms}$$

(3) $$Lopt\;\left(h_{rms},\theta ,VV\right)=\;1.281\;+\;0.134\;{\left(\sin0.19\;\theta \right)}^{-1.59}\;h_{rms}$$

where *θ* is expressed in degrees, and *Lopt* and *h_rms_* are expressed in cm. The formulations for *Lopt* (Equations (2) and (3)) were obtained with a Gaussian correlation function.

To generate a simulated dataset for the C-band (frequency = 5.045 GHz), the values for the parameters mentioned in Equations (2) and (3) are considered based on the values of the field samples (see Table 2). Consequently, (2–30 vol.%) with step of 0.1 vol.%, (0.1–5 cm) with 0.1 cm step, and (32–45°) with 0.1° step are used for moisture, *h_rms_* and *θ*, respectively. Each set of data in the wide data, including *h_rms_*, *ε_r_*, and *θ* as the inputs, and *σ*^0^ are generated by the CIEM. A similar approach, is considered for MDB, where instead of applying CIEM the wide data set based on the MDB model is generated.

The expressions of $\sigma_{VV}^{{}^{\circ}}$ and $\sigma_{VH}^{{}^{\circ}}$ of the modified Dubois model by Baghdadi et al. [36] are given by Equations (3) and (4):

(4) $$\sigma_{VV}^{{}^{\circ}}=10^{-1.138}{\left(\cos\theta \right)}^{1.528}\;10^{0.008\;\mathrm{cotan}\left(\theta \right)\;mv}\;{\left(kh_{rms}\right)}^{0.71\;\sin\left(\theta \right)}$$

(5) $$\sigma_{VH}^{{}^{\circ}}=10^{-2.325}{\left(\cos\theta \right)}^{-0.01}\;10^{0.011\;\mathrm{cotan}\left(\theta \right)\;mv}\;{\left(kh_{rms}\right)}^{0.44\;\sin\left(\theta \right)}$$

where *θ* is expressed in radians and *mv* is in vol.%. Equations (3) and (4) show that the sensitivity of the radar signal to the soil moisture in decibel scale is 0.10 dB/vol.% in VV polarization and 0.14 dB/vol.% in VH polarization for an incidence angle of 38° (SAR incidence angle for our study site). As for the signal’s sensitivity to soil roughness, it is twice as large in VV than in VH. The wide data sets simulated form CIEM and MDB are considered for NNs training.

### 3.4. Inverse Modeling of Soil Moisture Using an NNs

One common method for retrieving soil moisture is the NNs, which is used in this study. As explained previously, the CIEM and MDB models are applied to generate the simulated data. Actually, the main reason to create a simulated data is providing a rather reliable data by which the NNs can be well trained. In this case, the different NNs need to be separately trained for VV and HV polarizations. The structure of the neural networks is such that its input as well as the output is a two-dimensional vector. For our purpose, the input vector contains *σ*^0^ and *θ* values and the output vector contains the soil moisture values. It is worth noting that the feed forward NNs with TRAINLM as the training function is used in this study. In a neural network, a hidden layer is considered as a layer in between the input and output layers. In this layer, neurons are applied as weighted inputs and an activation function is used to create an output or a series of outputs. The NNs used in this study included 20 neurons in the hidden layer.

## 4. Results and Discussion

The results and discussion of this work are presented in two parts. In the first part, to evaluate the performance of the models, the extracted sigma naught as backscattering coefficients (*σ*^0^) from images are compared with the same parameters simulated from the models. In the second part, the feasibility of the neural networks (NNs) for estimation of the soil surface moisture, from Sentinel-1 images is investigated. During the field campaigns, 58 field parcels were visited to analyze a total of 142 measurements. For evaluation of the models, all data were employed. Moreover, for soil moisture estimation based on NNs, almost 70% of the measurements, namely 100 measurements, were randomly selected. Then, they were used as training data and the remaining 30% of the measurements, namely 42 measurements, were applied to test the accuracy of the model.

### 4.1. Comparison between the Images Extracted σ^0^ and the Model-Estimated σ^0^

As explained previously, to evaluate the accuracy of the selected backscattering models, at first the performance of two models, namely the calibrated IEM “CIEM” [17,18,19], and the modified Dubois model “MDB” [36] were examined.

#### Evaluation of the Models

**Figure 4:**
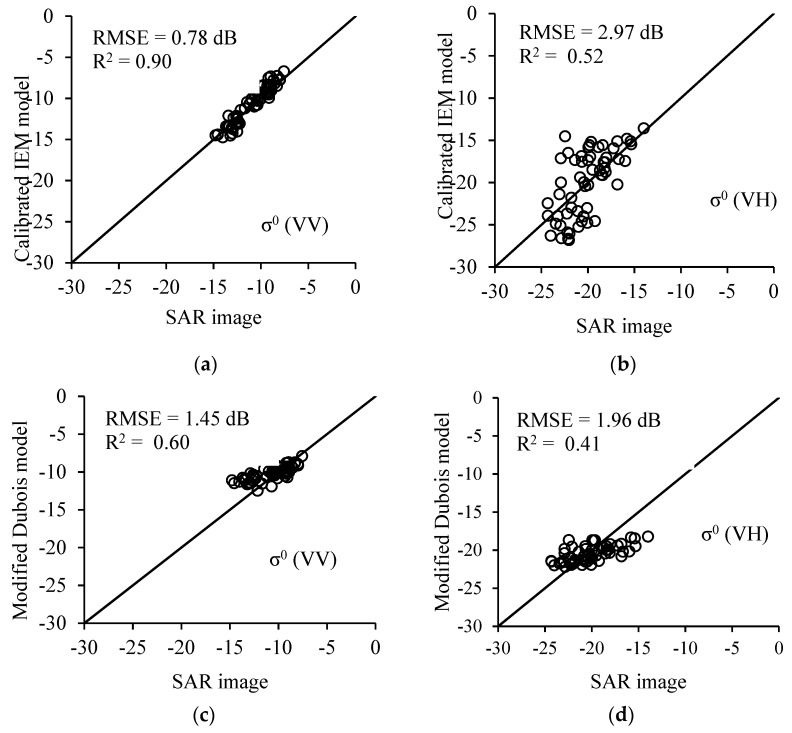
The relationship between the *σ*^0^ values extracted from synthetic aperture radar (SAR) images and the *σ*^0^ values estimated using the calibrated integral equation model (IEM) and modified Dubois models in VV and VH polarizations. (**a**) CIEM in VV; (**b**) CIEM in VH; (**c**) modified Dubois model (MDB) in VV; and (**d**) MDB in VH.

**Figure 5:**
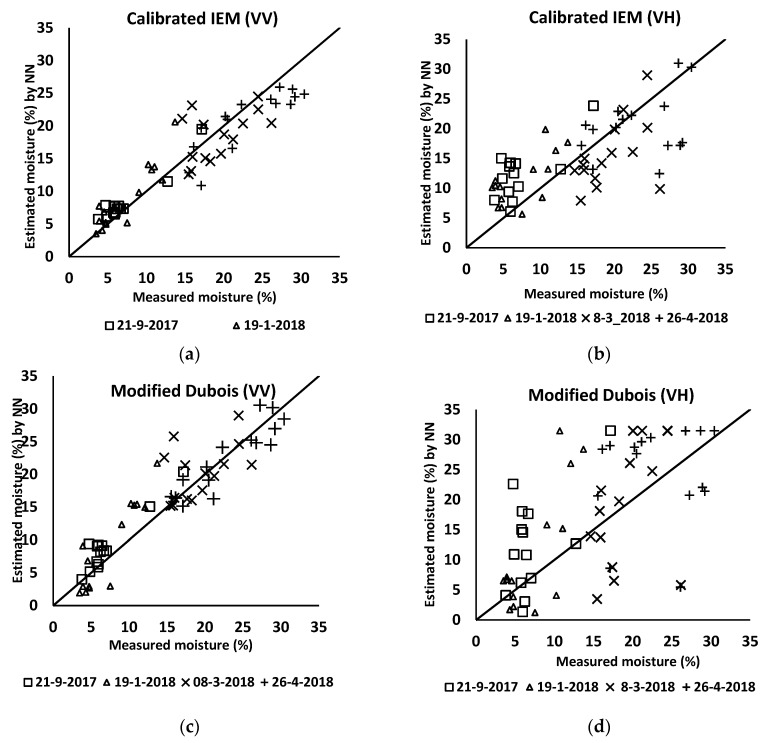
The relationship between the measured moisture (vol.%) vs. the estimated moisture (vol.%) using the neural networks. (**a**) CIEM with VV; (**b**) CIEM with VH; (**c**) MDB with VV; (**d**) MDB with VH.

Figure 4 shows the results of comparison between the *σ*^0^ values extracted from Sentinel-1 data and those estimated using the CIEM and MDB models in both channels of polarizations, namely VV and HV. Both models have a better performance in the VV polarization compared to the HV polarization. Moreover, for VV polarization, the performance of CIEM is better than that of MDB (RMSE of 0.78 for CIEM and 1.45 for MDB). The MDB overestimates by 2.1 dB the SAR data for low soil surface moisture (SSM) values (SSM < 10 vol.%). This was already observed in the Figure 4 and Figure 5 of the study of Baghdadi et al. [36]. The RMSE of the *σ*^0^ estimation in VV is 0.78 dB which is lower than that of VH (RMSE = 2.97 dB). However, for the MDB model, the obtained RMSEs are 1.45 and 1.96 dB for VV and VH polarizations, respectively. Thus, the CIEM model outperformed the modified Dubois model in *σ*^0^ estimation.

Figure 4 shows that for the VV polarization the SAR data fits better in CIEM than the MDB. In particular, MDB overestimates the SAR data for SSM lower than 10 vol.% (36% of available samples) (Figure 4c). To understand such observation, the relationship between SAR simulations in VV polarization and SSM for both CIEM and MDB was analyzed for an incidence angle of 35° and a *h_rms_* of 1.2 cm. Results showed that the relationship between SAR backscattering and SSM is logarithmic for CIEM and linear for MDB. Thus, the difference between CIEM and MDB simulations are observed mainly for SSM which is lower than 10 vol.%. Indeed, for SSM lower than 10 vol.% the MDB simulations well overestimates the CIEM simulations (by about 2 dB for SSM of 5 vol.%). This means that the CIEM simulates well the SAR backscattering corresponding to low SSM whereas the MDB probably overestimates the SAR backscattering for these same SSM-values. For VH polarization, similar observation is obtained as in the case of VV for SSM lower than 10 vol.%. However, for SSM higher than 25 vol.%, the MDB simulations at VH well underestimates the CIEM ones (by about 2.7 dB for SSM of 20 vol.%). Figure 4d clearly confirms that MDB overestimates the radar signal in VH for low SSM values and underestimates the radar signal for high SSM values.

### 4.2. Estimation of Soil Surface Parameters Using Neural Networks

The soil moisture was estimated based on the explanation provided in previous sections. To train the NNs, the simulated datasets were generated by applying the CIEM and MDB backscattering models: For each model, the simulated data were used to train NNs in each polarization (i.e., VV and VH) separately. Then, the trained NNs were employed to estimate the moisture surface of the field parcel polygon. Figure 5 compares the estimated moisture with those of the field measurements. Additionally, the values of RMSE and R^2^ resulted from the models are presented in Table 3. The RMSE for the CIEM model is 3.0 vol.% in the VV polarization. The same accuracy was obtained for the MDB model with an RMSE of 3.3 vol.%. As explained previously, 70% of the field measurements were applied for training data and the remaining 30% were used for the accuracy estimation.

According to Figure 5b,d, the RMSE of the estimated moisture when the MDB model with VH polarization was employed stayed at 8.8 vol.%. However, this value decreased to 5.9 vol.% when the CIEM model was used. The results indicate the high capability of the VV polarization when we use only one polarization.

## 5. Conclusions

The main objective of this research work was to assess the capability of bare soil moisture estimation over an agricultural area, namely Karaj (Iran), using Sentinel-1 images. To find the best correlation between SAR signal and soil moisture, two models (1) the calibrated IEM (CIEM) [21] and (2) the modified Dubois (MDB) [36] were formed and compared based on the real SAR observations and in situ soil moisture measurements. To reach the objective, in the first step, the backscattering coefficients in VV and VH polarizations simulated from the CIEM and MDB models were compared with those extracted from the Sentinel-1 images. The accuracy of the CIEM model (RMSE = 0.78 dB) was better than the MDB model (RMSE = 1.45 dB). In addition, both models presented more accurate results in VV polarization. The obtained results led to the conclusion that the CIEM with VV polarization performs with a satisfying accuracy.

In the second step, an inversion technique based on the neural networks (NNs) was developed to estimate the soil moisture from Sentinel-1 data. Then, soil moisture resulted from the NNs were compared with the ground truth measurements of the soil moisture. For this step, the RMSE on estimated soil moisture from VV polarization was 3.0 vol.% for the CIEM and 3.3 vol.% for the MDB. The results show two important conclusions: (1) The accuracy of soil moisture obtained for this study area can satisfy the requirement of the proposed method for soil moisture production; (2) the CIEM performs slightly better than the MDB in the study area.

## Figures and Tables

**Table 1:** Some weather data for field measurement dates.

Date (dd/mm/yyyy)	Solar Radiation (kWh/m^2^)	Temperature [min–max] (°C)	Daily Precipitation (mm)	Monthly Precipitation (mm)	Air Humidity (%)	Wind Speed m/s [min–max]	Visibility (Km)
21/09/2017	5.8	[15.1–31.4]	0	0	14	[5–7]	>10
19/01/2018	4.7	[4.0–13.5]	0	24	32	[8–22]	9
08/03/2018	4.8	[7.1–16.9]	0	18	34	[6–10]	9
26/04/2018	5.0	[12.6–23.2]	0	12	23	[7–16]	>10

**Table 2:** The field measurements and satellite images used in this study (Asc: Ascending, Des: Descending).

Date (dd/mm/yyyy)	Orbit	Incidence Angle θ (°) Over the Study Area [near–far]	# Field Samples	Moisture (%) [min-mean-max]	Soil Roughness (cm) [min–max]
21/09/2017	Asc	[37–38]	14	[2.19–10.83–17.7]	[0.64–2.77]
19/01/2018	Asc	[37–38]	14	[3.22–8.87–13.68]	[1.54–3.08]
08/03/2018	Asc	[37–39]	15	[14.62–20.79–26.12]	[0.64–3.43]
26/04/2018	Des	[37–39]	15	[15.45–23.71–30.65]	[0.64–2.54]

**Table 3:** The statistical parameters used to compare the measured field and estimated moisture (%) using the neural networks based on MDB and CIEM models with VV and VH polarizations.

			Moisture (vol.%) (21-9-2017)	Moisture (vol.%) (19-1-2018)	Moisture (vol.%) (8-3-2018)	Moisture (vol.%) (26-4-2018)	Moisture (vol.%) Full Series
CIEM	RMSE	VV	1.6	2.7	3.7	3.5	3.0
		VH	6.1	4.9	6.0	6.4	5.9
MDB	RMSE	VV	2.3	4.0	4.0	2.3	3.3
		VH	8.6	8. 6	8.9	9.2	8.8
